# Supplementary material for: Whole-exome sequencing of cell-free DNA and circulating tumor cells in multiple myeloma
Source: Nat Commun. 2018 Apr 27;9:1691. doi: 10.1038/s41467-018-04001-5 (PMC5923255; doi:10.1038/s41467-018-04001-5)

Whole-exome sequencing of cell-free DNA and circulating tumor cells in multiple myeloma

Manier & Park et al.

Supplementary Fig. 1. Graphical description of tumor biopsy DNA, cfDNA, and CTC DNA isolation from a patient. Magnetic beads selection steps were included for tumor biopsy and CTC DNA isolation to select CD138+ cells. Tiana Issa created the human stick figure, horseshoe magnet and DNA helix images via Adobe Photoshop.

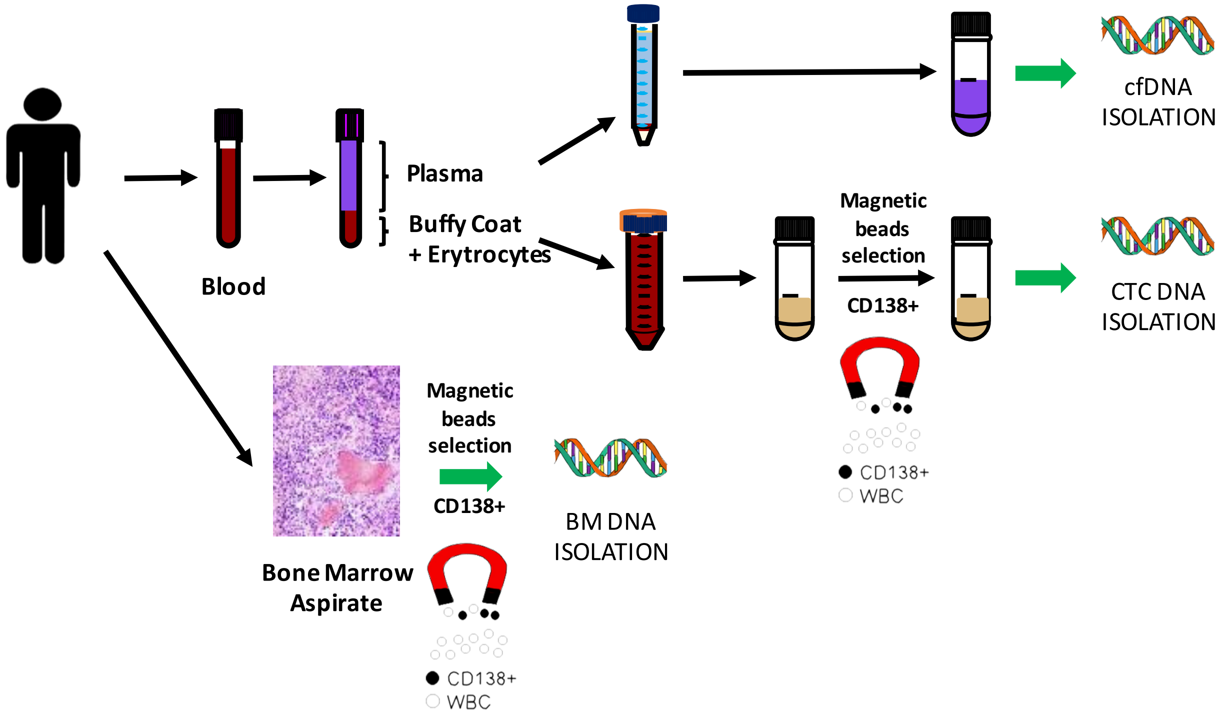

Supplementary Fig. 2. **(A)** Somatic mutation signatures in bone marrow biopsies, cfDNA, and CTC samples. Each color represents the base change (folded by strand, so mutations at reference G and T bases are reverse complemented), and each bar represents the frequency of a base change at a given mutational context with the indicated 5' and 3' bases. Y-axis represents counts for each base change. **(B)** Somatic mutation signatures in samples with only comparisons between bone marrow biopsies and cfDNA. **(C)** Somatic mutation signatures in the sample with only comparison between bone marrow biopsies and CTCs.

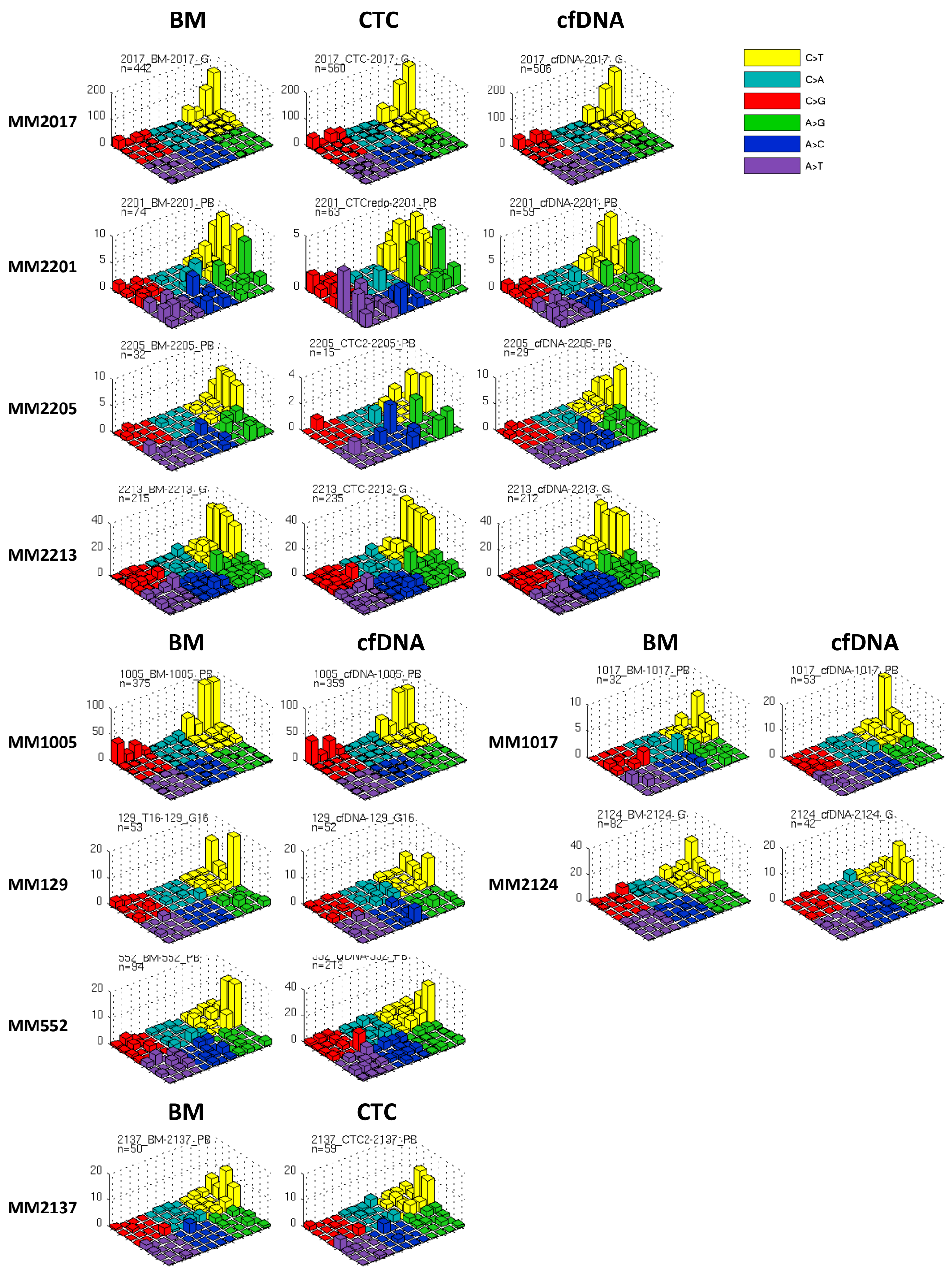

Supplement: Supplementary file 1 — Supplementary information [file 41467_2018_4001_MOESM1_ESM.pdf]
