# Supplementary material for: Whole-exome sequencing of cell-free DNA and circulating tumor cells in multiple myeloma
Source: Nat Commun. 2018 Apr 27;9:1691. doi: 10.1038/s41467-018-04001-5 (PMC5923255; doi:10.1038/s41467-018-04001-5)
Supplement: Supplementary file 3 — Description of Additional Supplementary Files [file 41467_2018_4001_MOESM3_ESM.pdf]

Description of Additional Supplementary Files

File Name: Supplementary Data 1

File Description: Clinical information of our cohort of patients provided as an Excel sheet.
